# Identifying Features of Electroencephalography Associated with Improved Awareness in Persistent Vegetative State via Multiscale Entropy: A Machine Learning Modeling Study

**DOI:** 10.1177/2689288X251369274

**Published:** 2025-08-27

**Authors:** Keyun Lai, Xiao Chen, Liyun He, Qi Liu, Changsheng Lai, Yang Bai, Ye Zhang, Kaiyue Wang, Fangzhao Wang, Shuai He, Guangjun Wang

**Affiliations:** ^1^ Xi’an Traditional Chinese Medicine Encephalopathy Hospital Affiliated to Shaanxi University of Traditional Chinese Medicine, Xi’an, China.; ^2^ Institute of Clinical Basic Medicine of Traditional Chinese Medicine, Chinese Academy of Chinese Medical Sciences, Beijing, China.; ^3^ Institute of Acupuncture and Moxibustion, China Academy of Chinese Medical Sciences, Beijing, China.; ^4^ Tianjin University of Traditional Chinese Medicine, Tianjin, China.; ^5^ Preventive Health Section, Yulin Red Cross Hospital, Yulin, China.

**Keywords:** electroencephalogram, machine learning, multiscale entropy, persistent vegetative state, prediction

## Abstract

Accurate differentiation between persistent vegetative state (PVS) and minimally conscious state and estimation of recovery likelihood in patients in PVS are crucial. This study analyzed electroencephalography (EEG) metrics to investigate their relationship with consciousness improvements in patients in PVS and developed a machine learning prediction model. We retrospectively evaluated 19 patients in PVS, categorizing them into two groups: those with improved consciousness (*n* = 7) and those without improvement (*n* = 12). Spectral and complexity analyses were performed on patients’ EEG data to obtain spectral power and multiscale entropy (MSE) values. These metrics served as features in developing an EEG-based prediction model for consciousness improvement. Spectral power and MSE values were used as features in six machine learning models—support vector machine (SVM), Classification and Regression Tree, chi-squared automatic interaction detector, neural network, C5.0, and logistic regression—to perform classification via data mining methods. The dataset, containing data of 19 cases, was divided into training and test sets at a 50% ratio. The SVM model using MSE features yielded the best classification results, with prediction accuracies of 95.18% (training set) and 92.93% (test set). The logistic regression model achieved 93.25% and 84.51% accuracy, respectively. In the test set, the MSE-based SVM model demonstrated a 27.67% improvement in classification accuracy compared with models using spectral analysis features, indicating that MSE achieves better classification performance. This study demonstrates that MSE is a promising predictor of prognosis in patients in EEG-confirmed vegetative states.

## Introduction

A vegetative state (VS) is a complex neurological disorder in which patients are unconscious of themselves and their environment, breathe spontaneously, have stable diurnal cycles, and exhibit behaviors that may mimic sleep and eye-opening.^1^ When VS lasts for more than 1 month, it is classified as a persistent vegetative state (PVS). Once PVS is diagnosed, the chances of recovery are considered low to moderate and are often associated with varying degrees of disability; in some cases, the condition lasts a lifetime. Higashi et al.^2^ reported an annual PVS incidence of 25/100,000, with global incidences increasing annually, positioning this condition as a major public health challenge. Currently, no specific drug or treatment has proven effective for PVS. The condition places a heavy burden on society and families due to its prolonged duration, high monitoring and treatment costs, and low rates of consciousness recovery.^3^ In addition, prognostic accuracy for patients in PVS raises serious ethical concerns, particularly when treatment decisions may include withdrawal of life support.^4^ For families of patients in PVS, questions about recovery likelihood and treatment pose significant emotional and ethical challenges.

Therefore, accurate and reliable outcome prediction is essential for PVS management. In the consciousness recovery process, the minimally conscious state (MCS) represents a necessary transition phase between VS and wakefulness. Numerous studies have shown that patients in PVS who progress to MCS have increased chances of further consciousness improvement and potential full wakefulness.^5,6^ Consequently, accurate differentiation between PVS and MCS in patients showing consciousness improvement is crucial for prognosis. This differentiation not only enables an early judgment before any change in condition, helping identify patients with greater therapeutic potential, but also largely influences treatment decisions and subsequent intervention strategies.

Despite its importance, accurately differentiating between PVS and MCS and estimating recovery likelihood in patients in PVS remain challenging.^7^ PVS diagnosis has traditionally relied on clinical assessments or subjective scoring systems such as the Coma Recovery Scale (CRS-R)^8^; however, these approaches have high misdiagnosis rates and often fail to accurately predict consciousness recovery.^7^ Although the global population of patients in PVS is increasing and research is advancing, there remains a critical shortage of validated objective prediction systems, which is a major challenge for society and patients’ families.

In recent years, researchers have explored objectivizing tools such as neuroimaging and neurophysiology techniques to improve diagnostic and predictive accuracy, with promising preliminary results.^9^ Among these methods, electroencephalography (EEG) offers particular advantages: it can be performed at the bedside and is relatively inexpensive, noninvasive, and repeatable. These qualities position EEG as a potentially valuable tool for predicting prognosis and consciousness recovery in patients in VS.

EEG detection techniques and analytical algorithms have advanced considerably.^10^ Classical EEG parameters such as spectral power can reveal brain states (waking or sleeping), whereas evoked potentials can detect certain cognitive processes. These methods include recording EEGs under auditory,^11^ visual,^12^ tactile,^13^ or mental stimuli.^14^ Early studies analyzing spectral power^10^ reported that compared with patients in MCS, those in VS exhibited increased delta power and decreased alpha power. However, spectral analysis methods have limitations in accurately processing the nonlinear and complex biological signal characteristics of conditions such as PVS.

EEG complexity analysis represents an innovative approach specifically developed for nonlinear and complex EEG signals. Several nonlinear dynamics algorithms have been applied to EEG analysis,^15^ with complexity^16^ and entropy^17,18^ being the most widely utilized. Feature-based EEG complexity methods include Lempel–Ziv complexity, approximate entropy (ApEn), cross-entropy, permutation entropy, and connectivity analysis.

EEG entropy, derived from thermodynamics,^19^ has been extensively applied to explore EEG signal features related to consciousness states in patients in VS.^20^ Nonlinear analysis of resting EEG using indices such as complexity and entropy has also been used in several studies^9,16,20–24^ to quantify the degree of consciousness in patients in a VS. Ma et al.^25^ assessed clinical resting-state EEG signals for consciousness detection metrics, demonstrating that certain metrics reliably reflect consciousness states across different wakefulness levels. Gosseries et al.^20^ correlated entropy values with CRS-R clinical assessment and healthy controls, finding that entropy values were higher in patients in MCS than in those in VS. Sara et al.^22^ reported that patients in VS with high ApEn values had a greater chance of improving and reaching MCS or even regaining full consciousness. Wu et al.^23^ found similar results to Thul et al.’s,^26^ where awake individuals showed higher entropy values and more deeply unconscious individuals exhibited lower values. These findings indicate that nonlinear EEG analysis can characterize brain function changes in unconscious states, with entropy values serving as potential indicators for predicting consciousness recovery in patients in VS.

Among these assessment methods, multiscale entropy (MSE) has emerged as a novel analytical approach. Compared with traditional entropy analysis methods, MSE extends the analysis to multiple time scales, enabling a more comprehensive evaluation of EEG signal complexity. Although EEG signals possess multiscale properties, ApEn can only analyze at a single scale, potentially missing critical information at particular scales—especially relevant conditions such as PVS that exhibit reduced complexity. The capability of MSE allows a deeper understanding of dynamic brain activity changes associated with PVS that might not be fully captured by single-scale analyses. By leveraging advantages of MSE, we aim to improve the accuracy of PVS prognosis prediction and potentially identify new biomarkers indicative of consciousness recovery. This research direction holds significant promise for advancing the understanding and management of patients in PVS.

In this study, we employed both EEG spectral and complexity analysis to explore key EEG indicators related to consciousness in patients in PVS. We extracted spectral power and MSE values as features to establish corresponding EEG signal features. Machine learning methods—including support vector machine (SVM) models, decision tree models (Click-Through-Rate and chi-squared automatic interaction detector [CHAID]), artificial neural networks, C5.0, and logistic regression—were used to build prediction models. To assess the discriminative power of the logistic regression model, we obtained the area under the curve (AUC) via the receiver operating characteristic (ROC) curve. The predictive accuracy was further evaluated using metrics including sensitivity and specificity.

## Methods

### Ethical approval and ethical opportunity

All patients were hospitalized at Xi’an Traditional Chinese Medicine Encephalopathy Hospital and Cerebral Diseases. This retrospective study was conducted following the Declaration of Helsinki and received approval from the Clinical Research Ethics Committee of Xi’an Hospital of Traditional Chinese Medicine and Cerebral Diseases. Patient confidentiality was maintained throughout the study to protect participants’ rights and interests.

### Participants

The inclusion criteria were as follows: patients (1) who were hospitalized at Xi’an Traditional Chinese Medicine Encephalopathy Hospital; (2) who were diagnosed with PVS based on seven criteria in the “Persistent Vegetative State” Diagnostic and Assessment Criteria revised at the 2011 Nanjing Conference,^27^ with the condition persisting longer than 1 month; and (3) who were assessed via two or more EEGs during hospitalization, with at least 20 days between consecutive recordings.

The exclusion criteria were: (1) patients with incomplete diagnostic and hospitalization records or adverse reactions occurring during the clinical study, making continuation inadvisable; (2) patients or families unwilling to continue observation and monitoring; (3) patients with fewer than two EEG examinations during hospitalization or <20-day interval between two EEG examinations; and (4) patients with complications occurring during EEG examination.

Patients were grouped according to the diagnostic criteria for PVS and the clinical efficacy rating scale developed at the 2011 Nanjing Conference,^27^ which has a maximum score of 20. A score of ≥12 on the PVS scale classified the patient as being out of VS, whereas a score of ≥6 classified the patient as being in an MCS.

By comparing clinical diagnostic results of patients’ PVS scores at admission and discharge, patients with a PVS score of ≥6 at discharge were categorized into the group with improved consciousness. Patients showing no improvement in consciousness level or signs of deterioration (PVS score below 6) or death were categorized into the group with no improvement in consciousness. This study tended to select cases with longer hospitalization and more comprehensive examinations, which may have introduced selection bias. To mitigate this bias, all participants meeting the inclusion criteria of having two EEG datasets were enrolled, regardless of hospitalization duration.

### Data sources and collection

Medical records meeting the above criteria from September 2021 to September 2023 were retrospectively screened. One patient was excluded because of epilepsy present during EEG assessment and poor data quality. Information from 19 patients was ultimately included.

**FIG. 1.**
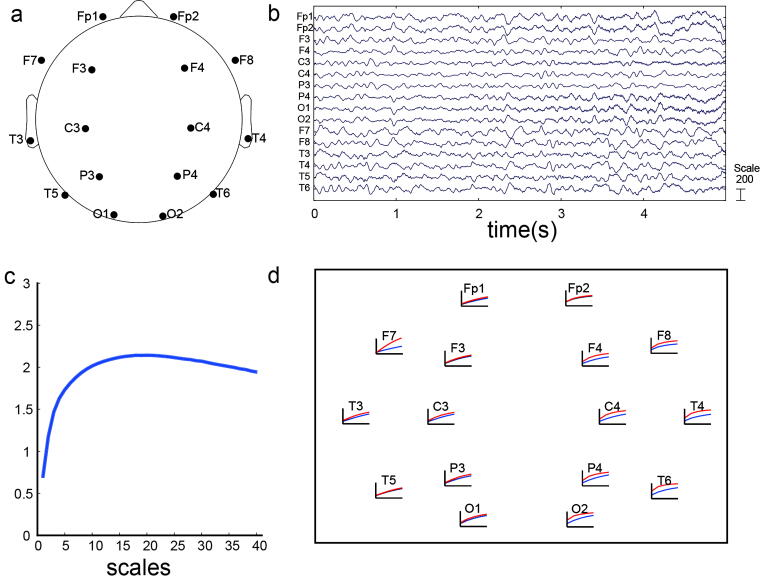
Electrode locations and examples of EEG signal and multiscale entropy profile. **(A)** Arrangement of EEG electrode placements according to the international 10–20 system; **(B)** Representative section of raw EEG data; **(C)** Changes in multiscale entropy values (scale = 40) on the P4 lead of a patient; **(D)** Multiscale entropy profiles across all leads in a representative patient; the blue and red curves represent the multiscale entropy values of the first and second EEG recordings, respectively. EEG, electroencephalography.

In this study, resting EEG was acquired using a 16-lead EEG signal detector of the Sun EEG machine (model: SOLAR6000B), with a sampling rate of 1,000 Hz and electrode impedance of <5 kΩ. Electrodes were placed according to the internationally recognized 10–20 system, with 16 recording electrodes and 1 reference electrode. Figure 1A shows the electrode placement. each patient’s brain wave data were collected for 24 to 25 h (except in special circumstances requiring <24 h), and raw EEG selections are shown in Figure 1B. During recording, the ward was maintained with natural lighting and free from excessive noise. All patients were placed in the supine position during data collection, with meals and treatments suspended.

### EEG data preprocessing

Raw EEG data were extracted in European Data format and imported into MATLAB software 2021b (MathWorks, Natick, MA, USA) for preprocessing. The data were bandpass filtered at 2–70 Hz, the sampling rate was reduced to 256 Hz, and bad segments were removed. A randomly selected 15-min segment of EEG signal from each case was used for analysis.

### Spectrum analysis

MATLAB was used to detrend the EEG data. The periodogram method was used to obtain a power spectrum that accurately reflected the original EEG, followed by the Welch method^28^ to estimate EEG signal power spectral density. The power spectrum was divided into five frequency bands: delta (0.3–3.5 Hz), theta (4.0–7.5 Hz), alpha (8.0–13 Hz), beta (14–30 Hz), and gamma (30–70 Hz).

For each patient, the total power and the power of each EEG frequency band were calculated separately for the first and second EEG recordings. The differences in total power and band-specific power were then obtained by subtracting the pretreatment (first) spectral power values from the posttreatment (second) values. In addition, the change in MSE values at the P4 lead was depicted as a curve [Fig. 1C]. The differences in total EEG power and in the power of the five frequency bands between the two recordings in patients with PVS were analyzed separately to determine whether these changes were associated with improvements in consciousness.

### Complexity of EEG

MSE is used to measure the complexity of EEG signals. Its theory was proposed by Costa,^29,30^ and the analysis method of the MATLAB toolbox was provided by PhysioNet.^31^ The 15-min EEG signals randomly selected as described above underwent complexity analysis with the following parameters: *N* = 96,000, *m* = 2, and *r* = 0.15. As shown in Figure 1C, MSE values (scale = 40) were calculated for all leads for each patient. Complexity at different scales served as the feature for classification, and the MSE value for each lead at 40 scales was obtained to form a 16 × 40 matrix. This matrix was then analyzed as the feature value for the input classifiers.

### Statistical analysis

Nonparametric comparisons were performed using the independent samples Mann–Whitney *U* test in SPSS 19.0 software. For spectral analysis, the difference between the total power values obtained was used as the test variable. A nonparametric test was performed to determine the difference between the total power of the spectral analyses before and after treatment, with the difference between the total power across 16 electrodes serving as the test variable and consciousness improvement status as the grouping variable. For MSE analysis, the AUC of entropy values at each electrode served as the test variable, with consciousness improvement status as the grouping variable. Differences were considered statistically significant at *p* < 0.05.

### Establishing prediction models

SPSS Clementine 12.0 was used to establish and train the classification and recognition models. The matrixes obtained from spectral and complexity analyses were imported into SPSS Clementine as a data source. Random sampling was performed via split nodes, with 50% of the dataset selected as the training set and the remainder as the test set. After the data were entered into the Clementine system nodes, the Select node was used to filter out the variables that entered the predictive model, and post-input and output variables were set in the Type field. Subsequently, the type of model (SVM, decision tree C5.0, and CHAID) was selected, and parameters were configured, initially using system default parameters. During this process, we adjusted several parameters, including changing the kernel function type from the default radial basis function (RBF) kernel to a polynomial kernel function, and left the rest of the parameters as default. The specifics of the names and values of each model parameter can be found in Table 1. The model node was activated to train the model according to the set parameters, generating the corresponding model upon completion. The generative model was connected to the above nodes to obtain the predictive model. Finally, the predictive model was connected to the analyze node for model analysis and evaluation.

**Table 1.** Names and Values of Each Model Parameter

Parameter table for SVM							
Parameter name	Stopping criteria	Regularization parameter（C）	Regression precision（epsilon）	Kernel type	Gamma	Bias	Degree
Value	0.001	10	0.1	Polynomial	1	0	3

Parameter table for CART			
Parameter name	Maximum surrogates	Minimum change in impurity	Impurity measure for categorical targets
Value	5	0.0001	Gini

Parameter table for CART			
Parameter name	Use partitioned data	Mode	Levels below root
Options	√	Generate model	5

Parameter table for CHAID					
Parameter name	Alpha for splitting	Alpha for merging	Chi-square for categorical targets	Epsilon for convergence	Maximum iterations for convergence
Value	0.05	0.05	Pearson	0.001	100

Parameter table for neural network										
Parameter name	Hidden layers	Layer 1	Layer 2	Layer 3	Persistence	Alpha	Initial Eta	High Eta	Eta decay	Low Eta
Value	3	20	15	10	200	0.9	0.3	0.1	30	0.01

Parameter table for C5.0			
Parameter name	Output type	Mode	Favor
Value	Decision tree	Simple	Accuracy

Parameter table for logistic regression			
Parameter name	Mode	Scale value	Singularity tolerance
Value	Simple	1.0	0.00000001

### Model evaluation

To assess the discriminatory power of the logistic regression model, the AUC was obtained from the ROC curve. The accuracy of the logistic regression model predictions was also evaluated using metrics such as sensitivity and specificity.

## Results

### Demographic and clinical characteristics

Among the 19 patients, 9 were male and 10 were female, with ages ranging from 26 to 68 years. Patients were divided into a consciousness improvement group (*n* = 7) and a consciousness nonimprovement group (*n* = 12). The mean age of patients in the consciousness improvement group was slightly higher than in the nonimprovement group (57.14 vs. 52.75 years), although there were no significant differences in consciousness improvement according to age, sex, or etiology. Detailed demographic and clinical data are presented in Table 2.

**Table 2.** Demographic and Clinical Characteristics of Study Participants

Characteristics	All participants
Age, mean (±SD) (year)	54.37 (±2.734)
Sex	
Male, *n* (%)	9 (47.37)
Female, *n* (%)	10 (52.63)
Etiology	
Cerebral hemorrhage, *n* (%)	13 (68.42)
Contraindication, *n* (%)	5 (26.32)
Subarachnoid hemorrhage, *n* (%)	1 (5.26)
Clusters	
Consciousness improvement, *n* (%)	7 (36.84)
Improved from VS to awareness	2 (10.53)
Improved from MCS to awareness	1 (5.26)
Converted from VS to MCS	3 (15.79)
Converted from VS to MCS+	1 (5.26)
Awareness not improved, *n* (%)	12 (63.16)
Maintaining a vegetative state	9 (47.37)
Maintaining a minimally conscious state	1 (5.26)
Death	2 (10.52)

MCS, minimally conscious state; SD, standard deviation; VS, vegetative state.

### Spectral analysis

Table 3 shows the differences in spectral power values, along with *p*-values and statistics related to the consciousness state obtained via nonparametric tests. Results revealed that differences in total spectral power values at leads FP2, F4, P3, P4, and F8 significantly differed between patients with and without consciousness improvement.

**Table 3.** Spectral Power Differences Between Patient Groups and Associated Statistics

Channel	Awareness improvement group (10^4^ μV^2^/Hz)	Consciousness nonimprovement group (10^4^ μV^2^/Hz)	*z*-Values	*p*-Values
FP2	−0.54 (−3.39, 0.94)	0.95 (−0.65, 8.46)	−2.484	0.013
F4	−0.30 (−2.57, 0.57)	0.22 (−0.68, 3.32)	−2.361	0.018
P3	−0.16 (−0.90, 0.21)	0.11 (−0.17, 1.89)	−2.438	0.015
P4	−0.10 (−1.21, 0.41)	0.05 (−0.22, 2.02)	−2.029	0.042
F8	−0.56 (−13.62, 0.86)	0.55 (−0.86, 4.88)	−2.561	0.01

Values are expressed as medians (interquartile range) (M [P25, P75]).

After categorizing spectral power values into five frequency bands—alpha, beta, delta, theta, and gamma—nonparametric tests were performed on the differences between spectral power values of each frequency band from the two EEG recordings. The power values for both groups at C3 and P4 leads across each band are presented in Table 4, with statistically significant *p*-values highlighted in bold. Only the C4 and P3 leads in the alpha band showed significant differences related to consciousness improvement. No significant differences were observed between electrodes in the remaining frequency bands. The statistical analysis results comparing alpha wave activity with other frequency bands at C4 and P3 leads relative to consciousness improvement are also shown in Table 4.

**Table 4.** Power Values and Statistical Comparisons for C3 and P4 Leads Across Five Frequency Bands

Bands	Channel	Awareness improvement group (10^4^ μV^2^/Hz)	Consciousness non improvement group (10^4^ μV^2^/Hz)	*z*-Values	*p*-Values
Alpha	C4	−0.27 (−1.06, −0.18)	0.18 (−0.19, 1.82)	−2.028	**0.043** ^*^
	P3	−0.50 (−1.30, 0.00)	0.07 (−0.33, 1.11)	−2.028	**0.043** ^*^
Beta	C4	0.002 (−0.74, 0.17)	0.08 (−0.04, 0.41)	−1.268	0.205
	P3	−0.10 (−0.43, −0.01)	0.002 (−0.06, 0.29)	−1.69	0.091
Delta	C4	−0.287 (−11.08, 10.20)	2.81 (−5.00, 5.04)	−0.676	0.499
	P3	1.03 (−17.21, 22.09)	1.425 (−3.21, 7.94)	−0.761	0.447
Gamma	C4	−0.057 (−0.20, 0.48)	0.004 (−0.13, 0.40)	−0.845	0.398
	P3	−0.16 (−0.35, 0.20)	0.034 (−0.09, 0.33)	−1.69	0.091
Theta	C4	0.26 (−1.40, 2.06)	−0.66 (−2.89, 2.44)	−0.845	0.398
	P3	0.49 (−1.99, 1.82)	1.02 (−2.16, 2.89)	−0.338	0.735

Values are expressed as medians (interquartile range) (M [P25, P75]).

^*^ *p* < 0.05.

### MSE analysis

The change in area under the EEG entropy curve for a representative patient across two recordings is depicted in Figure 1D. The blue and red curves represent the MSE values of the first and second EEG, respectively. Table 5 shows the correlation between changes in area under the MSE curve and consciousness improvement. In the improved consciousness group, the area under the entropy curve of the second EEG compared with that of the first decreased in four patients, increased in two patients, and remained unchanged in one patient. In the nonimprovement consciousness group, five patients showed a decrease in area, whereas seven patients showed an increase. Fisher’s exact test indicated (*p* = 0.251) no significant correlation between changes in area under the entropy value curve and consciousness improvement (Table 5).

**Table 5.** Correlation Between Changes in Area under the Multiscale Entropy Curve and Consciousness Improvement

Change in area under the entropy curve	Awareness improvement group	Consciousness nonimprovement group	X^2^-values	*p*-Values
Entropy increases	2 (10.5)	7 (36.8)	2.765	0.251
Entropy decreases	4 (21.1)	5 (26.3)		
Entropy remains unchanged	1 (5.3)	0		

Values expressed as *n* (%).

Furthermore, in nonparametric testing using the area under the entropy value curve for each electrode as the test variable, only the P4 channel showed a significant association with consciousness improvement (*p* = 0.036), with no significant differences observed at other electrodes.

### Establishment of the prediction models

Table 6 shows the classification accuracy of machine learning models using spectral power values as features. Classification accuracy ranged from 60% to 85%, with linear models (such as logistic regression) achieving higher accuracy than nonlinear models. Results demonstrated that across models evaluating both entropy values and consciousness state in patients in PVS, those using MSE as the feature value produced the best classification outcomes, regardless of whether they employed linear logistic regression or nonlinear machine learning approaches.

**Table 6.** Classification Accuracy of Machine Learning Models Using Spectral Power Values as Features

Features	Type	Model	Accuracy (%)
Spectral analysis	Nonlinear model	SVM	65.26
		CART	82.11
		CHAID	58.95
		Neural network	63.16
		C5.0	83.16
	Linear model	Logistic regression	84.21

SVM, support vector machine; CART, Classification and Regression Tree; CHAID, chi-squared automatic interaction detection.

As shown in Table 7, the highest accuracy among all machine learning prediction models using MSE features was 95.18%, achieved by the SVM model in the training set. Other prediction models also produced satisfactory classification results. Similarly, Table 8 shows that the best classification accuracy of machine learning models using MSE features in the test set was 92.93%. Thus, as shown in Tables 6 and 7, among all classifiers used in this study, SVM performed the best in distinguishing consciousness state improvement in patients in PVS. Furthermore, comparing Tables 5 and 6 shows that in most cases, classification results using MSE as features outperformed those using spectral power values.

**Table 7.** Classification Accuracy of Machine Learning Models Using MSE as Features in the Training Set

Features	Type	Model	Accuracy (%)
MSE	Nonlinear model	SVM	95.18
		C5.0	88.75
		CHAID	87.46
		Neural network	90.03
		Bayes Net	91.64
		CART	87.78
	Linear model	Logistic regression	93.25

**Table 8.** Classification Accuracy of Machine Learning Models Using MSE as Features in the Testing Set

Features	Type	Model	Accuracy (%)
MSE	Nonlinear model	SVM (polynomial)	92.93
		C5.0	81.14
		CHAID	79.8
		Neural network	88.22
		Bayes Net	67.68
		CART	80.13
	Linear model	Logistic regression	84.51

MSE, multiscale entropy.

### Model evaluation

**FIG. 2.**
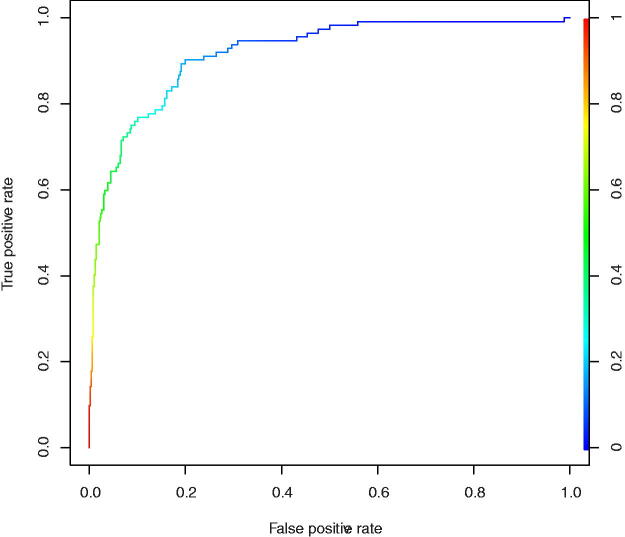
Logistic regression model ROC plot. ROC, receiver operating characteristic.

A valuable visualization tool for comparing classification models is the ROC curve (Table 9), where sensitivity is plotted as the vertical coordinate and specificity as the horizontal coordinate. The AUC measures model accuracy, with values ranging from 0.5 to 1. The closer the AUC is to 1, the better the model prediction. The SVM model achieved 92.93% prediction accuracy in the test set, with 73.21% sensitivity and 97.51% specificity. The positive predictive value in the test set was 87.23%, and the negative predictive value was 94%. Where the ROC plot for the logistic regression model is shown in Figure 2, the accuracy, sensitivity, and specificity of the remaining models are shown in Table 9.

**Table 9.** MSE Classification Performance Metrics for Each Prediction Model in Training and Testing Sets

Model	Collect	AUC	Sensitivity (%)	Specificity (%)
SVM	Training	0.863	75.00	99.60
	Testing	0.812	73.21	97.51
C5.0	Training	0.701	41.07	99.22
C5.0	Testing	0.589	23.21	94.60
CHAID	Training	0.728	50.00	95.69
CHAID	Testing	0.629	35.71	90.04
Neural network	Training	0.758	53.57	98.04
Neural network	Testing	0.756	55.36	95.85
Bayes Net	Training	0.783	64.29	97.65
Bayes Net	Testing	0.629	61.24	93.23
CART	Training	0.739	32.14	95.85
CART	Testing	0.710	41.17	95.85
Logistic regression	Training	0.831	69.64	97.25
Logistic regression	Testing	0.793	55.36	91.29

AUC, area under the curve.

## Discussion

In the present study, MSE emerged as the most relevant indicator of consciousness state improvement. Among the six predictive models evaluated, the SVM model using MSE features demonstrated the best predictive performance in terms of classification accuracy and model evaluation metrics. Learning models characterized by MSE more accurately differentiated patient prognosis, whereas classification accuracy using spectral power values was generally lower than that achieved with MSE.

### Adjustment of kernel function

The kernel function maps original data to a higher dimensional space to identify the optimal classification surface. Different mapping methods substantially influence classification effectiveness. After adjusting the kernel function from RBF to polynomial, classification accuracy improved in both test and training sets. This appears to be a key factor in enhancing classification accuracy. The polynomial kernel function involves multiple parameters, whereas RBF typically has only one parameter. By adjusting multiple parameters, the polynomial kernel function can better adapt to data characteristics and improve model performance. Although the RBF kernel has fewer parameters, parameter selection has marked effects on model performance.

### Spectral analysis for the PVS prediction study

Consistent with our findings, previous studies on spectral analysis have demonstrated that alpha and theta bands are more involved in human cognition,^32^ and alpha band power can differentiate between patients in VS and those in MCS.^33,34^ Compared with patients in MCS, those in VS typically show reduced alpha power and increased delta band power. Regarding connectivity, both theta and alpha bands were significantly lower in patients in VS than those in MCS.^35^ Similarly, Stefan et al.^9^ found that alpha frequency power was greater in patients in MCS than in those in VS, supporting the validity of using alpha spectral power for prediction. Furthermore, a recent clinical study evaluating neural characteristics of resting-state EEG in 380 individuals showed that relative power in alpha and delta bands can serve as reliable consciousness indicators.^25^ This finding suggests that alpha band power likely represents a key indicator associated with consciousness improvement in patients in VS.

However, three prediction models using spectral power values in this study achieved accuracies below 70%. This may be related to the limitations of most spectral analysis methods, which can only obtain overall signal spectra without local analysis capability, making them more suitable for deterministic smooth signals.^36^ Therefore, we further employed complexity analysis to improve classification accuracy in predicting PVS outcomes.

### Application of MSE in EEGs

Although spectral analysis has gained increasing attention in EEG research, complexity analysis better aligns with the nonlinear nature of physiological signals. EEG complexity represents the most reliable marker of consciousness state.^16^ Previous studies have shown that high and low entropy values can distinguish between patients in PVS and those in MCS.^16,20^ Patients with high entropy demonstrate relatively better consciousness states, whereas those with low entropy show poorer consciousness states,^23,26^ indicating that reduced entropy values may be associated with loss of consciousness-related complexity. This study preliminarily explored correlations between entropy value changes and consciousness improvement, although no definitive conclusions were reached, possibly due to the small sample size.

MSE provides a unique method for observing nervous system complexity across various time scales. Although MSE application in PVS prognosis remains limited, it has been applied to diagnosis and prediction of various neurological disorders, including epilepsy, Alzheimer’s disease, and Parkinson’s disease. The combination of power spectral density with MSE could serve as a biomarker for distinguishing between ictal and interictal epileptiform patterns in patients with typical catatonic seizures, potentially facilitating seizure prediction.^37^ This finding suggests value in combining spectral analysis with complementary methods to improve prediction accuracy. Similarly, MSE is widely used in the diagnosis of Alzheimer’s disease.^38,39^ Machine learning methods have successfully differentiated between healthy controls; mildly cognitively impaired individuals; and mild, moderate, and severe patients with Alzheimer’s disease.^40^ Results indicate a high correlation between Alzheimer’s disease severity and MSE, with reduced complexity reflecting diminished information processing ability in patients with Alzheimer’s disease. This categorization by disease severity better reveals potential components of neurological disease progression, although finer groupings may reduce classifier performance. Therefore, categorical predictions of PVS, MCS, and wakefulness must be carefully considered in patients in PVS classification studies. In addition, MSE can analyze EEG features during sleep in patients with Parkinson’s disease.^41^ These studies demonstrate MSE’s utility for analyzing complex EEG signals, providing a foundation for integrating MSE into clinical practice as a reliable prognostic tool for PVS.

MSE characterizes EEG signal complexity across time scales, allowing subdivision of EEG data into distinct temporal resolutions. In analyzing EEGs from patients in PVS as well as from those with epilepsy, Alzheimer’s disease, and Parkinson’s disease, underlying patterns and appropriate time scales are often unknown. Complexity analysis of EEG signals at different scales using MSE effectively extracts information from EEG data by providing richer observational perspectives, better capturing dynamic complexity of EEG signals, and more accurately revealing temporal changes. In addition, when processing large volumes of EEG data, MSE effectively prevents overfitting and facilitates dimensional reduction of data, improving analytical accuracy. This could be the reason for MSE classification achieving higher accuracy than spectral analysis in our study.

However, owing to sample size limitations, this study did not differentiate between etiological factors such as whether PVS resulted from traumatic brain injury or cerebrovascular disease. Moreover, randomly selecting 15-min EEG segments reduced analytical complexity but meant that raw data were not fully utilized. Therefore, future studies should include multicenter, large-sample, prospective studies to validate the accuracy of the predictive models. This will allow for a more precise assessment of their usefulness, facilitate testing and refinement, and ultimately confirm their validity.

## Conclusion

In this study, EEG spectral analysis and complexity analysis were used as research methods to explore the EEG features potentially related to consciousness improvement in patients in PVS. The results showed that the SVM model featuring MSE demonstrated superior prediction performance in terms of classification accuracy and model evaluation, suggesting that MSE is a promising indicator for prognosis in patients in PVS. The underlying mechanisms of MSE’s predictive capability need to be further explored.

## Data Availability

The datasets generated during the current study are available from the corresponding author upon reasonable request.

## Abbreviations Used

ApEn: approximate entropy
AUC: area under the curve
CART: Classification and Regression Tree
CHAID: chi-squared automatic interaction detector
CRS-R: Coma Recovery Scale
EEG: electroencephalography
MCS: minimally conscious state
MSE: multiscale entropy
PVS: persistent vegetative state
RBF: radial basis function
ROC: receiver operating characteristic
SVM: support vector machine
VS: vegetative state
